# Intervention impact on depression product appraisal and purchasing behavior by employers: a randomized trial

**DOI:** 10.1186/1472-6963-14-426

**Published:** 2014-09-24

**Authors:** Kathryn M Rost, Donna Marshall, Stanley Xu

**Affiliations:** Department of Mental Health Law and Policy, College of Behavioral and Community Studies, University of South Florida, 13301 Bruce B. Downs Boulevard, Tampa, FL 33612 USA; Colorado Business Group on Health, 12640 West Cedar Avenue, Suite A, Lakewood, CO 80228 USA; Institute for Health Research, Kaiser Permanente, 10065 E. Harvard Avenue, Suite 300, Denver, CO 80231 USA; Department of Biostatistics and Informatics, School of Public Health, University of Colorado Denver, Denver, USA

**Keywords:** Depression care management, Employers, Return on investment, Academic detailing, Implementation science, Randomized trial

## Abstract

**Background:**

Employers can purchase high quality depression products that provide the type, intensity and duration of depression care management shown to improve work outcomes sufficiently for many employers to achieve a return on investment. The purpose of this randomized controlled trial was to test an intervention to encourage employers to purchase a high quality depression product for their workforce.

**Methods:**

Twenty nine organizations recruited senior health benefit professional members representing public or private employers who had not yet purchased a depression product for all 100+ workers in their company. The research team used randomization blocked by company size to assign eligible employers to: (1) a presentation encouraging employers to purchase a high quality depression product accompanied by a scientifically-derived return on investment estimate, or (2) a presentation encouraging employers to work with their most subscribed health plan to improve depression treatment quality indicators. Two hundred ninety three employers (82.3% of 356) completed baseline data immediately before learning that 140 employers had been randomized to the evidence-based (EB) depression product presentation and 153 had been randomized to the usual care (UC) depression treatment quality indicator presentation. Analysis of 250 (85.3% of 293) employers who completed web-based interviews at 12 and/or 24 months was conducted to determine presentation impact on depression product appraisal and purchasing behavior.

**Results:**

The intervention had no impact on depression product appraisal in 232 subjects (F = 2.36, p = .07) or depression product purchasing (chisquare = 1.82, p = .44) in 250 subjects. Depression product appraisal increased in companies with greater health benefit generosity whose benefit professionals were male. Depression product purchasing behavior increased in small companies compared to large companies, companies who knew a vendor that sold depression products at baseline, companies with greater health benefit risk taking, and companies with less politicalization of health care benefit decision making.

**Conclusions:**

Policy makers need to build innovative bridges to the employer community to convince them to purchase evidence-based benefits, even when benefits offer potential financial savings.

**Trial registration:**

Clinical Trials Registration Number: NCT01013220.

## Background

Policy analysts have long recognized that five stakeholder groups (purchasers, plans, practices, providers, and patients) have to actively support efforts to enhance primary care depression treatment for sustainable gains to be realized [1]. Multiple randomized trials [2, 3] and demonstration projects [4, 5] have been conducted to build this support among plans, practices, providers, and patients with comparatively little effort focused on purchasers. Purchaser support is vital because models that improve outcomes [2, 3] also increase the direct costs of care [6], at least for the first year a depressed individual participates [7].

In the U.S., employers represent a substantial segment of purchasers, offering health insurance coverage to 88.8% of individuals in the workforce [8]. In addition, employers have ‘a skin in the game’. Studies demonstrate that 7.6% of U.S. employees suffer a major depressive episode each year [9] which substantially reduces their capacity to work as evidenced by increased absenteeism [10] and reduced productivity at work [10, 11]. Randomized trials demonstrate that interventions which improve depression treatment increase work functioning [12] so that selected employers can realize a return on investment (ROI) [13] with competitively priced evidence-based products [14]. Earlier studies note that employers report substantial information deficits about the costs that organizations absorb from employee depression, and note that they are willing to apply program savings from improved absenteeism and productivity at work against program costs [15].

In a study of health benefit professionals representing 293 U.S. employers across the country interested in depression in the workplace, we conducted a randomized trial to test an intervention which provided employers a scientifically derived company-specific ROI estimate with the purchase of a high quality depression product. Provided by health plans, disease management vendors and managed behavioral health organizations, depression products provide screening, education, systematic monitoring, and clinician feedback for all interested employees at an additional cost to the health premium [14]. In the first randomized controlled trial to our knowledge to be conducted in health benefit professionals, the study hypothesized that employers randomized to the intervention condition would report more positive depression product appraisal and greater depression product purchasing behavior over 24 months than employers randomized to the comparison condition.

## Methods

### Design

**Figure 1:**
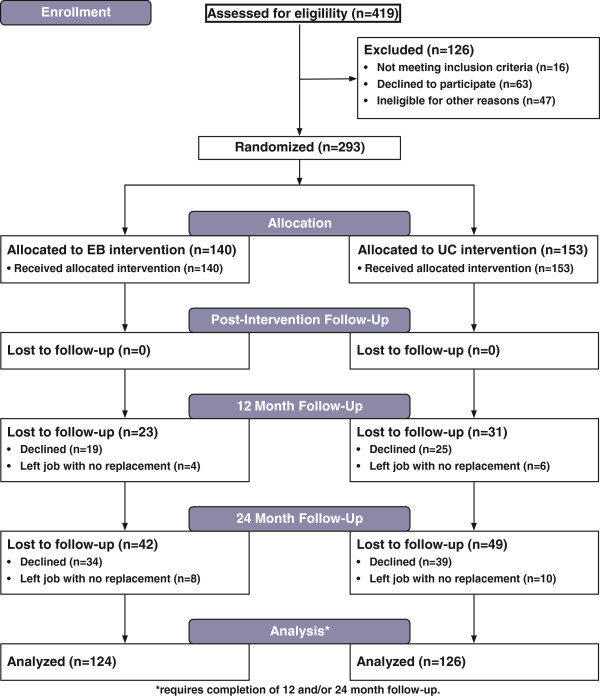
**Flow diagram.**

To conduct this randomized controlled trial, the research team modified its previously published design [16] to invite all National Business Coalition on Health (NBCH) coalitions and 12 related professional groups in the United States to participate in the study to recruit the number of needed subjects. The 29 coalitions/groups responding to the invitation reviewed eligibility criteria before inviting interested senior health benefit professionals (hereafter referred to in this manuscript as employers) to participate in the study. Eligibility criteria required that interested employers: (a) represent public or private companies employing 100 or more non-unionized employees, (b) their companies intended to remain in the coalition/group for the next two years, and (c) their companies had not already purchased a depression product for all its employees. In both the proposed and implemented design, the study excluded unionized employees because unions generally negotiate health benefits for their members directly with employers. Identified employers were block randomized by size within coalition at a 1:1 ratio to one of two conditions between April 2009 and May 2011, when recruitment was terminated to provide sufficient time for two year follow-up. Employers in each condition were invited to participate in an intervention or comparison presentation held in their coalition/group’s geographic area to learn more about state-of-the-art strategies employers could utilize to improve depression in the workplace. Two hundred ninety three of 356 identified employers (82.3%) met eligibility criteria and attended the presentation (or webinar at a later date) to which they had been randomized. Baseline data were collected immediately before employers learned which strategy their presentation addressed. Follow-up data were collected immediately after the presentation, at 12 months, and at 24 months (see Figure 1), with the final subject completing follow-up in July 2013. The protocol and the informed consent that employers signed were approved by the Institutional Review Boards at Florida State University and the University of South Florida.

Presentation - Described in detail [16, 17], employers randomized to the intervention condition received an evidence-based (EB) presentation, while employers randomized to the comparison condition received an enhanced usual care (UC) presentation. Based on academic detailing interventions [18, 19], both presentations presented scientifically-based information on depression in the workplace. Both presentations were delivered by the same presenter in separate meetings (e.g., one in the morning and the other in the afternoon). Email reminders of key points in the presentation plus encouragement to request free unbiased technical assistance from the presenter were sent 15-, 17-, and 19-months after the presentation. The presenter (DM) was a former NBCH board member, who herself directed a regional business coalition which participated in pilot testing the intervention. The presenter completed a survey immediately following each presentation verifying that presentation components were delivered according to protocol.

Intervention (EB) Presentation – Piloted and refined before its implementation, the 25 minute EB presentation educated employers about high quality depression care and its evidence-based impact on clinical and work outcomes [12, 20]. Each participant received a company-specific ROI generated by a calculator that the research team developed in earlier studies translating scientifically derived estimates of the impact of high quality depression care on absenteeism and productivity at work to a monetized savings in lost work days, varying pertinent employee, organizational, and vendor characteristics [21]. Employers who did not provide the information needed to calculate an ROI before the presentation were provided ROIs by email as soon as they provided the data to the research team. The presenter explicitly encouraged employers to explore purchasing a high quality depression product and offered free unbiased technical assistance.

Comparison (Enhanced UC) Presentation - A similar length presentation educated employers about Healthcare Effectiveness Data and Information Set (HEDIS) indicators for antidepressant medication management and their use in monitoring outpatient depression treatment quality. Employers received HEDIS indicators for antidepressant medication management for their most subscribed plan if that plan reported its HEDIS scores to the National Committee for Quality Assurance (NCQA); otherwise, employers received HEDIS depression indicators for all plans in the area. During the presentation, employers were asked to encourage their most subscribed health plan to improve its HEDIS indicators for depression (or to calculate its HEDIS indicators if it did not report them) and offered free unbiased technical assistance. Because previous studies indicate that the information included in the HEDIS presentation will have little to no impact on employer health benefit purchasing [22–24], purchasing behavior in the enhanced UC condition captures vendor efforts to market depression products to employers in both conditions during follow-up.

### Data collection

Employers completed computerized surveys immediately before and after the presentation in the presentation room and at 12 and 24 month follow-up at their home or office. The research team member who actively contacted employers who did not respond to an email cue to complete follow-up was blinded to experimental condition. Employers were paid $100 for completing the pre- and post-presentation survey, $100 for completing 12-month follow-up, $100 for completing 24-month follow-up, and an additional $50 bonus for completing all surveys or for completing the 24-month follow-up survey if they failed to complete the 12-month survey. EB and UC employers were comparably likely to complete 12 and/or 24 month follow-up (see Figure 1).

### Operationalization of major constructs

Depression Product - Each survey defined depression product as a “depression disease management program to assure all employees with depression have the opportunity to get high quality care for the condition by confidentially providing education, monitoring and clinician feedback. By depression product, we do not mean a disease management program for chronic illness that advertises it provides care management for depression to the physically ill employees it serves.”

Depression Product Appraisal - We defined product appraisal as the ratio between perceived organizational benefit of a depression product and its relative organizational cost. Higher appraisal values reflect higher benefits at lower costs. Perceived organizational benefit was measured as the mean of four items (alpha = 0.84) that employers completed after they read the following vignette. “Imagine that your organization purchased a depression product. By depression product, we mean [insert definition from above]. The program costs your organization $800/year for each participating employee. You know two other companies who told you they thought that the product was worthwhile.” The four items are: (a) Would you expect the product to help depressed worker meet responsibilities at work over the short term (the first 6 weeks)?; (b) Would you expect the product to help depressed worker meet responsibilities at work over the long term (the first 6 months)?; (c) Would you expect the product to help prevent friction between depressed worker and his/her coworkers?; (d) Would you expect the product to help reduce treatment costs that contribute to increase in health premiums the next year? Response choices were: (1) no help; (2) little help; (3) moderate help; (4) considerable help; (5) great deal of help. Relative organizational costs was measured by a single item. “Managers often face the following issues when an organization considers a new health program. How would you rate the program described above?” with reverse-coded response choices being (1) much worse than programs our organization has recently undertaken; (2) worse than …; (3) better than …; and (4) much better than programs our organization has recently undertaken. If an employer reported that a depression program would provide them little help in helping workers meet their short term (Question a) and long term responsibilities (Question b) at work, moderate help preventing friction between the depressed worker and his/her coworkers (Question c), and no help reducing treatment costs (Question d), the numerator of the ratio would be calculated as 2 + 2 + 3 + 1 = 8. If an employer reported that that a depression program costs were much better than programs the organization had recently undertaken, the (reverse coded) denominator would be 2. The depression product appraisal ratio would be 8/2 = 4.

Depression Product Purchasing Behavior –We measured purchasing behavior of a depression product over the previous 12 months by employer self-report using a four-level ordinal variable: (1) no internal or external discussion of a depression product and no product pursuit; (2) internal or external discussion and no product pursuit, (3) internal and external discussion and no product pursuit, and (4) product pursuit. Internal discussion was defined as an email exchange, telephone call, in-person meeting or group meeting with other employees of the company. External discussion was defined as an email exchange, telephone call, in–person meeting or group meeting with a vendor. Product pursuit was defined as decision to purchase a depression product, release of a request for proposal (RFP) for a depression product, or completion of a contract with a vendor for a depression product. This definition of product pursuit captured several employers who met eligibility criteria for the study because they had released an RFP but had not completed a contract at baseline.

Wellness Product Purchasing Behavior – Because the study was designed to understand health benefit decision-making, employers were asked to identify other benefits they pursued in the 24 months following the presentation by asking employers at 12- and 24-month follow-up “In your organization, what new health benefits/initiatives were given priority this year?” Employers who responded to this open-ended question were allowed to identify more than one benefit/initiative. A research team member developed 13 categories (e.g., employee assistance program, healthy baby program) plus other before coding all responses to the question at 12-month follow-up [25]. A second coder used the categories to code all responses to the question at 24-month follow-up. To measure wellness product purchasing behavior (yes/no), we identified those employers who indicated they had prioritized a wellness, health rewards and/or prevention product (not otherwise specified) at 12 and/or 24 months. We chose to examine wellness benefits because our coalition collaborator [DM] indicated that wellness had become a ‘hot’ topic in benefit purchasing.

Covariates – We examined the contribution of baseline measures of organizational characteristics, health benefits characteristics, employer characteristics, and methods characteristics to intervention impact [26, 27]. Organizational characteristics included organizational size, age, type, number of sites, absenteeism monitoring, presenteeism (productivity at work) monitoring, centralized health benefit decision making, local health benefit decision making, size of health benefit purchasing group, financial member in health benefit purchasing group, and NBCH affiliation. Health benefit characteristics included perceived depression impact, estimated depression prevalence in workforce, number of health plan carriers, insurance risk, health benefit generosity (defined in Table 1), any mental health carveout, any employee assistance program (EAP), health benefit risk taking (defined in Table 1), resources for new health benefits in the next year, expected percentage increase in health insurance premiums, politicalization of health care benefit decision making (defined in Table 1), knowledge of vendor who sells depression products, and company-specific estimate of ROI with a depression product (provided to EB employers and calculated for enhanced UC employers). Characteristics of the employer (e.g., the senior health benefit professional in the company who provided all data on the company in this study) included gender, race/ethnicity, age, and report of his/her influence in company’s health benefit decision making. Methods characteristics included employer completion of intervention in group presentation versus webinar, original employer versus replacement employer completion of follow-up interview, and intervention fidelity. Intervention fidelity was defined as presenter checklist that all components of the presentation had been completed.

**Table 1:** **Measurement of key covariates**

Construct	Coding of items	Content of items
Health Benefit Generosity	Sum of 11 benefits to which employers contributed some or all of the costs	Employee Assistance Programs
		Return to Work Programs
		Chronic Disease Management Programs
		Stress Reduction Programs
		Smoking Cessation Programs
		Obesity Programs
		Prenatal or Well Baby Programs
		Grief Recovery Programs
		Fitness Facilities or Membership
		Onsite Site Vaccinations
		Health Risk Appraisals
Health Benefit Risk Taking	Mean of responses to 5 items coded on a 4 point Likert scale	1. Our organization’s health benefits philosophy is that in the long run we get ahead playing it slow, safe and sure (reverse coded).
		2. Our organization has built its health benefits program by taking calculated risks at the right time.
		3. Decision-making about health benefits in our company is too cautious for maximum effectiveness (reverse coded).
		4. Health benefits managers in our organization are willing to take a chance on a good idea.
		5. It is necessary to take some pretty big risks occasionally to keep our health benefits ahead of our competitors.
Politicalization of Health Benefit Decision-Making	Single item	In most organizations, some individuals have more influence than others in benefit decision-making. For example, one person may make a final decision without looking for substantial input because s/he is in a position where people are expected to make final decisions (influence because of position). Alternatively, one person can influence a final decision because the decision-maker particularly values his/her opinion (influence because of “who you know”). During the past 12 months, were differences in influence in benefits decision-making in your organization due to differences in:
		1. position primarily
		2. position more than “who you know”
		3. “who you know” more than position
		4. “who you know” primarily

Contamination – Because employers were randomized within coalitions, it is possible that employers randomized to enhanced UC learned about depression products from their colleagues in the coalition who were randomized to the EB condition. To examine this, the research team created a variable for enhanced UC subjects only that indicated the average post-presentation depression product appraisal value reported by EB colleagues with whom the UC subject had professional contact with during the previous year.

### Data analysis

The research team used a linear mixed model to test intervention impact on appraisal and a longitudinal ordinal model to test intervention impact on purchasing behavior while taking into account the dependence of observations over time and within coalition. The longitudinal ordinal model tested intervention impact without assuming a linear effect over time, reflecting that employers would be expected to report lower purchasing values at 24-month follow-up if they purchased a depression product at 12-month follow-up. Depression product appraisal was log transformed to achieve a normal distribution before analysis. Covariates that predicted the dependent variable at p < .20 were included in the final models testing intervention impact. The research team analyzed all available data over 24 months (allowing employers who completed either 12- and/or 24-month follow-up to remain in the analysis), testing intervention impact using two-tailed p values. The contamination analysis used the linear mixed models described above to examine the impact of EB colleague appraisal ratings on UC appraisal ratings in enhanced UC subjects only. Sensitivity analyses of intervention impact on product appraisal and purchasing behavior were conducted in employers who completed both follow-ups and analyses using last value carried forward. Depression product purchasing behavior at baseline was included as a covariate in the appraisal model and as the first wave of the dependent variable in the depression product purchasing models. Changes from baseline in appraisal and purchasing behavior measures were obtained from these statistical models and compared between EB and enhanced UC groups. The 250 employers who completed either or both follow-ups provided the study 80% power using two-tailed p values to find a .40 effect size on depression product purchasing behavior with an intraclass correlation as high as 0.02 introduced by the clustering of subjects within coalitions. Exploratory analyses were conducted by examining the frequencies of alternative benefits that employers prioritized in the two years following the presentation.

## Results

Description – Randomization produced EB and enhanced UC employer groups with statistically comparable organizational, health, subject and methods characteristics (see Table 2) in 32 comparisons with the exception that EB employers were more likely to be racial/ethnic minorities.

**Table 2:** **Baseline characteristics**

	Overall	EB Group	Enhanced UC group
	(n = 293)	(n = 140)	(n = 153)
***Organizational characteristics***			
Number of U.S. (SD) sites	23.4 (114.0)	32.6(156.7)	15.0 (47.2)
Size			
% small (100 to 500 employees)	34.1	30.7	37.3
% medium (501 to 2500 employees)	30.4	30.0	30.7
% large (2501 plus employees)	35.5	39.3	32.0
Type			
% for-profit	57.0	53.6	60.3
% not-for-profit	21.0	23.6	18.5
% public sector	22.0	22.8	21.2
Company age (SD)	75.9 (47.9)	76.8 (50.1)	75.0 (45.0)
% with any absenteeism monitoring	73.2	74.1	72.4
% with any productivity at work monitoring	56.4	53.8	58.7
Mean size of health benefit purchasing group (SD)	7.1 (6.4)	7.2 (6.5)	7.0 (6.4)
% centralized decision making	93.8	95.0	92.8
% local decision making	85.6	89.2	82.3
% purchasing groups with finance representative	80.1	78.5	81.7
% National Business Coalition on Health (NBCH) member	72.7	71.4	73.9
***Health benefit characteristics***			
Mean depression impact (SD) *	2.4 (0.5)	2.5 (0.5)	2.4 (0.5)
% estimating depression prevalence greater than or equal to 11%	51.8	50.8	52.8
Mean number of health plan carriers (SD)	2.2 (2.5)	2.3 (2.9)	2.1 (2.1)
Insurance risk			
% fully insured	21.5	23.9	19.3
% self-insured	48.3	46.4	50.0
% mixture of full and self-insured	30.2	29.7	30.7
Health benefit generosity	6.3 (3.0)	6.4 (2.9)	6.2 (3.1)
% with any mental health carveout	18.2	20.8	18.7
% with Employee Assistance Program (EAP)	80.4	80.6	80.3
Mean health benefit risk taking (SD) *	2.3 (0.5)	2.3 (0.5)	2.4 (0.5)
Mean new health benefit resources (SD) *	2.5 (0.7)	2.5 (0.7)	2.6 (0.7)
Mean expected % premium increase (SD)	7.4 (5.2)	7.8 (5.1)	6.9 (5.2)
Mean politicalization of health care benefit decision-making (SD)*	1.6 (0.8)	1.6 (0.8)	1.6 (0.9)
Mean estimated return on investment with depression (SD)	2.5 (2.0)	2.5 (2.1)	2.4 (2.0)
% knowledge of any vendor who sells depression products	50.0	51.8	48.3
% previous pursuit of depression product at baseline (SD)	1.4 (0.8)	1.4 (0.8)	1.5 (0.9)
***Employer characteristics***			
% female	69.8	71.2	67.9
% racial/ethnic minority^+^	13.6	17.1	9.2
Median age	41-50 years	41-50 years	41-50 years
% moderate to complete influence over benefit decision-making	74.5	76.4	73.2
***Methods characteristics***			
% completing presentation in person	84.1	85.0	83.0
% replacement subject completed 12 and/or 24 month follow-up	5.0	7.9	2.2
% presentation fidelity	100	100	100

*Scale of 1–4 with higher scores representing greater amounts of the construct.

^+^p < .05.

*SD* = standard deviation.

**Figure 2:**
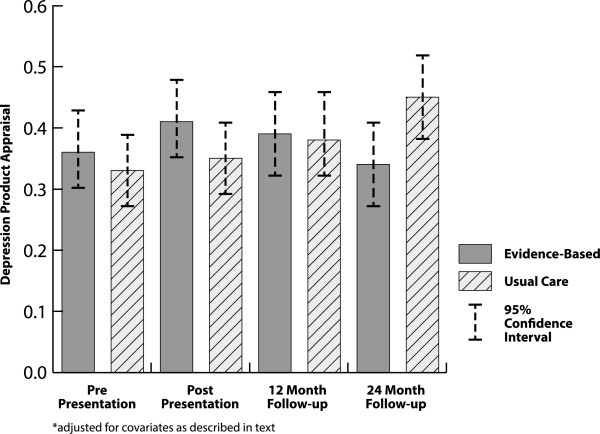
Intervention impact* on (Log) depression product appraisal.

At baseline, EB employers reported a non-transformed depression product appraisal ratio of 1.56 (SD = 0.65) while enhanced UC employers reported a 1.46 (SD = 0.48) ratio. At baseline, EB employers reported a 1.34 (SD = 0.75) depression product purchasing score while enhanced UC employers reported a 1.48 (SD = 0.87) score.Depression Product Appraisal – Depression product appraisal values could be estimated for 232 of the 250 subjects who completed 12 and/or 24 month follow-up, with 18 subjects eliminated from analysis because of missing data. The intervention had no impact on depression product appraisal over 24 months (ES = .03, F = 2.36, p = .07) in the covariate adjusted model (see Figure 2).

**Figure 3:**
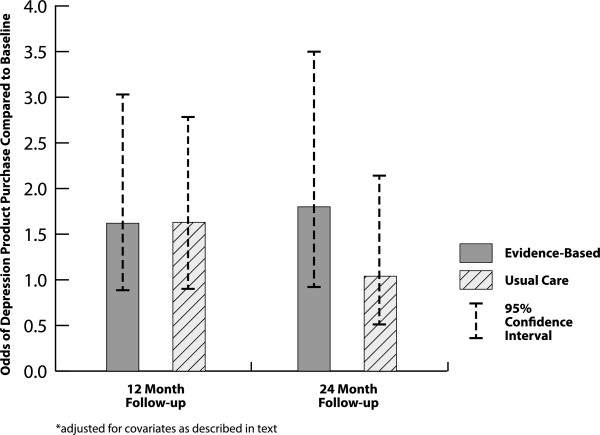
Intervention impact* on depression product purchasing behavior.

Covariates meeting inclusion criteria for the final appraisal model included gender, previous knowledge of vendor, absenteeism monitoring, presenteeism monitoring, health benefit risk taking, health benefit generosity, organizational need, and previous depression product purchasing activity. In the final appraisal model, increasing depression product appraisal over time was independently predicted by companies whose participants were male (beta = .08, p = .03) and by greater benefit generosity (beta = .01, p = 03). Estimated ROI or its interaction with intervention did not predict change in depression product appraisal over time in bivariate analyses and thus was not included in the final model. Sensitivity analyses in employers who completed both follow-ups and analyses using last value carried forward also failed to show significant intervention impact.Depression Product Purchasing Behavior – Depression product purchasing behavior could be estimated for all 250 subjects who completed follow-up. The intervention had no impact on depression product purchasing behavior over 24 months (chisquare = 1.82, p = .44) in the covariate-adjusted model (see Figure 3). Organizational characteristics meeting criteria for inclusion in the final model included organizational size, age, benefit generosity, health benefit risk taking, absenteeism monitoring, and financial member in health benefit making decision group. Health benefit characteristics included politicalization of decision, insurance risk, EAP, previous knowledge of depression vendor, mental health carveout and expected benefit premium increase. Employer covariates included influence on health benefit decision making in company.

In the final depression product purchasing model, greater odds of purchasing behavior were independently predicted by company size (small companies more than large companies, beta = -0.58, p = .03), previous knowledge of a vendor that sold depression products (beta = 1.23, p < .0001), greater health benefit risk-taking (beta = 0.60, p = 0.03), and less politicalization of health care benefit decision making (beta = -0.56, p = .0004). Estimated ROI or its interaction with intervention did not predict depression product purchasing behavior over time in the bivariate model and thus was not included in the final model. Sensitivity analyses in employers who completed both follow-ups and analyses using last value carried forward also failed to show significant intervention impact.

Contamination – The contamination variable was not related to depression product appraisal change over 24 months in enhanced UC employers in bivariate analysis (F = 1.01, p = .46); thus, multivariate analyses were not undertaken.

Exploratory Analysis – Two hundred two of the 250 employers completed the open-ended question about benefits that were given priority at either 12- and/or 24-months, with 41.8% of EB employers reporting that wellness programs were given priority in their companies in the previous year compared to 36.5% of enhanced UC employers (chi square = 0.59, p = .44).

## Discussion

This study demonstrated that brief intervention presenting scientifically derived company-specific ROI estimates associated with purchase of a high quality depression product delivered by a nationally recognized employer coalition director had no impact on employer appraisal or purchasing behavior of depression products over two years. Depression product appraisal was more likely to increase in companies whose participants were male and companies with greater benefit generosity. Depression product purchasing behavior had greater odds of increasing in small companies than large companies, companies who knew a vendor that sold depression products at baseline, companies whose cultures supported greater health benefit risk taking, and companies with less politicalization of health care benefit decision making. It is difficult to compare these findings to previous research because the research team identified only one other intervention study currently in progress [28] to influence decision-making in public health benefit purchasers.

If in fact policy researchers learn more from their failures than from their successes, this trial produced hard-won insights. Although it was considerably more challenging to identify interested coalitions and employers than our pilot study indicated, the recruitment protocol resulted in bringing influential health benefit professionals “to the table” with 75% of employers reporting moderate to complete influence over health benefit decision-making in their company. The project explicitly designed its recruitment strategy to study ‘ready to change’ employers rather than a nationally representative sample to observe non-diluted intervention impact. Despite the fact that participating employers initially reported substantial interest in and capacity to adopt depression products [26], their failure to request any free and unbiased technical assistance indicated they were not ‘ready to change’. Participating employers were however ‘ready to be studied’, as standard follow-up protocols achieved generalizable follow-up rates allowing 85% of subjects to remain in longitudinal analyses. The high follow-up rate is particularly notable because no other published study has conducted longitudinal research in a national sample of health benefit decision-makers, providing the country’s first data on the attitudes and behaviors of employer purchasers and how they change over time in response to and independently of intervention. We note that this study does not directly inform the field’s understanding of health benefit decision-making in countries with national health programs where employers are not direct purchasers of care.

The intervention tested in this trial was modeled after a presentation piloted in 15 employers in the presenter’s (DM) coalition, which resulted in 20% of participating employers pursuing depression products over two years. Since both the pilot study and the intervention trial presented similar content to presumably similar employers, we considered three different explanations for the pilot study’s success and the intervention trial’s failure to influence purchasing behavior: (1) historical differences, (2) design differences, and (3) leadership support differences.

Historical Differences – Because the intervention trial directly coincided with the initiation of health care reform which required health benefit professionals to focus their attention on new federal requirements, it is possible that health care reform reduced the success of the intervention. While the competing demands of health care reform increased the study’s difficulty recruiting coalitions and employers, reform did not completely stall health benefit decision-making as ~40% of EB employers pursued new wellness/prevention programs in the two years following the presentation. With modest scientific evidence of their ROI on health care expenditures [29], wellness programs appear to have moved into the employer spotlight that depression enjoyed in the mid 2000’s when the current intervention trial was planned. Employer choice to pursue wellness programs over depression disease management reflects that employers may be more enthusiastic about programs which promise to deliver ROI in observable reductions in health care expenditures rather than difficult to observe improvements in workplace productivity. Employer choice to pursue wellness programs over depression disease management may also reflect that employers prioritize ‘plug and play’ benefits likely to reach the broadest number of employees, moving depression and other mental health conditions far down the list.

The intervention trial also coincided with increasing employer disillusionment with disease management initiatives in general [30] as purchasers recognized that vendors often failed to deliver programs with the promised reductions on health care expenditures. In retrospect, the enthusiasm we observed about disease management programs in the pilot study had molted to serious skepticism by the time of the intervention trial. While disappointing, employer skepticism towards disease management products is not unfounded. After learning that only one of 14 depression product vendors sold a product delivering the four critical components of depression disease management at the recommended intensity/duration [14], the research team expanded the intervention to provide free technical consulting to employers on how to purchase a ‘high quality’ depression disease management product. Sadly, the one vendor selling a competitively-priced high quality product discontinued it after three years for lack of demand.

Design Differences – The pilot study utilized a pre-post intervention-only design to test the EB presentation, while the intervention trial randomized employers to the EB or enhanced UC conditions. While the enhanced UC presentation did not provide any information on depression products and their potential for ROI, the instruments that both EB and enhanced UC employers completed pre- and post-presentation provided a detailed description of depression products to ensure that all participants referred to the same product when rating it. While the instrumentation may have caused more similar between-group appraisals than would have otherwise existed, it is unlikely that the instrumentation influenced enhanced UC employers to purchase depression products (were it that easy…). A second source of design error was the potential for content sharing between employers in the same coalition randomized to opposite presentations; however, we could observe little evidence of this. In balance, the decision to randomize within coalition to reduce the impact of historical threats appeared to have worked in the study’s favor.

Leadership Support Differences – Another critical difference between the pilot study and the intervention trial was the support that the directors of participating coalitions/groups provided. In the pilot study, the coalition director (DM) presented critical parts of the content to employers in her coalition, emphasizing to employers who trusted her that researchers had succeeded in defining the critical components of a purchasable depression product to improve workplace outcomes. In the intervention trial, coalition directors had substantially less involvement, oftentimes balancing the demands of the study against the needs of other initiatives the coalition was prioritizing. Many employers in the intervention trial refused to accept that scientific estimates of workplace productivity attributable to depression were generalizable to their company, citing that before they would purchase a depression product they would need hard evidence of productivity deficits in their own workforce [25] rather than accept rigorously derived national estimates. Other employers questioned the study’s ‘real’ intention, suspicious that the research team was working with Accountable Care Act proponents to ‘sell’ employers on health care reform. To increase employer’s trust in the science, future intervention scientists may need to design interventions that can be co-delivered by a nationally recognized experts and local opinion leaders (e.g., coalition directors or leading employers in the coalition).

Limitations in the study’s design and implementation could also have contributed to the study’s negative results. We selected a single presenter to provide both the EB and enhanced UC presentations to assure that presenter characteristics did not inadvertently contribute to the outcomes we observed. In retrospect, we recognize that our objective fidelity measure could not capture whether the presenter (DM) was perceived as favoring one approach to improving depression treatment more than the other. Further research is needed to investigate this and other sources of bias in the effective presentation of evidence-based benefits to employer audiences. It is also possible that our study measures were not sufficiently sensitive to change, particularly our measure of depression product appraisal. While measurement in any new field can no doubt be improved, we do not think instrumentation challenges made a major contribution to this negative trial. Analyses conducted from both a classical test theory and item response theory approach demonstrated that the organizational benefits construct (the numerator in depression product appraisal) had encouraging psychometric validity [26]. Our initial examination of this measure showed that it significantly predicted product pursuit in the pilot trial.

## Conclusions

This study contributes to the literature by describing the disappointing outcomes of the first randomized trial to encourage health benefit professionals to purchase value-based health care products with ROI. The intervention had no observable impact on depression product appraisal or product purchase over two years. The lackluster employer response to relevant scientific findings presented in an easy to understand manner with award-winning graphics documents that the chasm between implementation science and health benefit decision-makers is huge. Policy makers will need to build innovative bridges to employers before these purchasers trust and utilize findings from implementation science, even when the directions the science recommends indicate the potential for financial savings.

## Abbreviations

EAP: Employee assistance program
EB: Evidence-based
HEDIS: Healthcare effectiveness data and information set
NBCH: National Business Coalition on Health
ROI: Return on investment
UC: Enhanced usual care.
